# The mediating roles of self-efficacy and professional identity in the association between internship environment and voice behavior among nursing interns: a multicenter cross-sectional study

**DOI:** 10.3389/fpsyg.2026.1879452

**Published:** 2026-10-02

**Authors:** Tao Xiao, Wei Cui, Meimei He, Yixia Deng, Jia Guo, Yayun Xiang

**Affiliations:** 1 Clinical Nursing Teaching and Research Section, The Second Xiangya Hospital, Central South University, Changsha, Hunan, China; 2 Department of Hematology, The Second Xiangya Hospital, Central South University, Changsha, Hunan, China; 3 Department of Geriatrics, The Second Xiangya Hospital, Central South University, Changsha, Hunan, China

**Keywords:** internship environment, mediation analysis, nursing interns, professional identity, self-efficacy, voice behavior

## Abstract

**Aim:**

To examine the association between the internship environment and voice behavior among nursing interns and to explore the statistical indirect effects involving self-efficacy and professional identity.

**Design:**

A multicenter cross-sectional study.

**Methods:**

From January to February 2026, 498 nursing interns from hospitals in Hunan Province, China, completed questionnaires on internship environment, self-efficacy, professional identity, and voice behavior. Descriptive statistics, Pearson correlation analysis, and regression analysis were conducted. PROCESS Model 6 with 5,000 bootstrap samples was used to estimate the individual and combined indirect effects involving self-efficacy and professional identity.

**Results:**

Internship environment was positively correlated with voice behavior (*r* = 0.553, *p* < 0.001), self-efficacy (*r* = 0.498, *p* < 0.001), and professional identity (*r* = 0.538, *p* < 0.001). After adjustment for demographic and internship-related characteristics, the 95% bootstrap confidence intervals for the individual indirect effects involving self-efficacy and professional identity and the combined indirect effect involving both variables did not include zero for overall, promotive, or prohibitive voice behavior. The descriptive ratios of the estimated total indirect effect to the estimated total effect were 55.98% for overall voice behavior, 49.47% for promotive voice, and 62.48% for prohibitive voice.

**Conclusion:**

A more supportive internship environment was associated with higher levels of overall, promotive, and prohibitive voice behavior among nursing interns. Significant indirect effects involving self-efficacy and professional identity were also observed in these associations. Clinical educators should consider fostering psychologically safe and supportive internship environments in which nursing interns feel able to express suggestions and patient safety concerns.

## 1 Introduction

The global nursing shortage is a prominent problem (Penturij-Kloks et al., 2023). Nursing interns are an important reserve force in the nursing workforce (Zhang et al., 2024). During internship, nursing students enter real clinical settings and begin to participate in patient care under supervision (Almadani et al., 2024). Effective communication and the willingness to express suggestions are essential for nursing interns when participating in clinical practice (Bradbury-Jones et al., 2011). However, nurses often hesitate to express their ideas. Limited clinical experience, hierarchical workplace structures, and fear of negative evaluation may discourage them from speaking up (Bradbury-Jones et al., 2011; Morrow et al., 2016). Therefore, it is important to understand factors associated with voice behavior among nursing interns.

Voice behavior refers to the voluntary expression of suggestions, ideas, or concerns that aim to improve organizational functioning (Lee et al., 2024). It is a form of proactive behavior because it challenges existing practices and promotes constructive change. Previous studies have shown that voice behavior has been associated with lower burnout, greater psychological safety, and more innovative behavior (Lee et al., 2024; Pundt, 2024; Lainidi et al., 2025). Liang further divided voice behavior into two types (Liang et al., 2012). Promotive voice focuses on proposing new ideas and improvements. Prohibitive voice focuses on pointing out risks, mistakes, or harmful practices. Both forms are important in healthcare settings (Schwappach and Niederhauser, 2019). Studies show that nursing students frequently notice patient safety concerns during clinical practice but do not always report them (Hoffmann et al., 2022). Silence in such situations may increase safety risks and limit opportunities for organizational improvement. Therefore, it is important to understand what encourages nursing interns to express their concerns and suggestions.

Social Cognitive Theory proposes that behavior is shaped by reciprocal interactions among environmental conditions, personal cognitive factors, and behavior (Bandura, 2001). The internship environment may influence nursing interns' voice by shaping both their perceived capability and professional self-regulation. Supportive supervision, constructive feedback, successful clinical experiences, and role modeling can provide important sources of self-efficacy (Pu et al., 2025). Stronger self-efficacy may increase interns' confidence, persistence, and willingness to accept the interpersonal risks of speaking up (Carrillo et al., 2024). Through observational learning and professional socialization, the internship environment may also help interns internalize nursing values and responsibilities, thereby strengthening professional identity (Peng et al., 2025; Yuan et al., 2025). Self-efficacy therefore represents the belief that one can speak up effectively, whereas professional identity provides a reason why one should speak up for patient safety and quality improvement. From this perspective, voice behavior reflects the joint contribution of environmental support, perceived capability, and internalized professional responsibility. In this study, the internship environment represents the environmental context, while voice behavior represents the behavioral response. Self-efficacy reflects interns' perceived capability to speak up, whereas professional identity reflects their internalized professional values and responsibilities. Together, these factors explain how environmental support may be associated with nursing interns' voice behavior.

Existing evidence suggests that nursing interns' voice behavior reflects both environmental support and personal readiness. A supportive clinical learning environment may strengthen professional identity through self-efficacy (Gao et al., 2022), while professional identity is also associated with nursing interns' patient safety attitudes (Zhao et al., 2024). Studies of speaking up further indicate that psychological safety and perceived impact are important to voice behavior among nurses and nursing students (Lee et al., 2024; Hoffmann et al., 2022). Taken together, these findings suggest that the internship environment may provide opportunities and support for speaking up, self-efficacy may strengthen interns' confidence to express their views, and professional identity may reinforce their responsibility to protect patient safety. Thus, self-efficacy reflects whether interns believe they can speak up, whereas professional identity reflects whether they believe they should speak up.

The internship environment provides the context in which nursing students integrate theoretical knowledge with practical experience (Flott and Linden, 2016). Studies have shown that positive internship environments are associated with higher learning satisfaction, stronger professional competence, and better psychological well being among nursing students (Mansutti et al., 2017). Self-efficacy is a central concept in Social Cognitive Theory. It refers to an individual's belief in their ability to perform tasks successfully (Bandura, 2001). People with higher self-efficacy usually show stronger initiative and persistence when facing challenges (Klassen and Klassen, 2018). Interns who feel confident about their abilities may be more willing to engage in voice behavior. Professional identity is another important psychological factor during nursing internship. Professional identity refers to the internalization of professional values, responsibilities, and roles (Worthington et al., 2013). Nursing interns who strongly identify with their profession may feel responsible for patient safety and quality improvement (Zhao et al., 2024). This sense of responsibility may encourage them to express concerns or suggestions when problems occur.

Previous research has linked the clinical learning environment to nursing students' professional identity through self-efficacy (Gao et al., 2022; Cai et al., 2022). However, it did not examine voice behavior as an outcome. Research on nursing students' speaking up has mainly focused on patient safety concerns (Hoffmann et al., 2022), without explaining the roles of self-efficacy and professional identity. Thus, it remains unclear how the internship environment is related to nursing interns' promotive and prohibitive voice through these psychological factors. Social Cognitive Theory offers a useful framework for addressing this gap. A supportive internship environment may provide mastery experiences, constructive feedback, and role modeling, which may strengthen self-efficacy. Stronger self-efficacy may also support the development of professional identity. Integrating these relationships may provide a more comprehensive explanation of nursing interns' voice behavior.

Therefore, this study aimed to examine the relationship between internship environment and voice behavior among nursing interns. This study also examined the individual and combined indirect effects involving self-efficacy and professional identity based on SCT. Voice behavior includes promotive voice and prohibitive voice. Both dimensions were considered in the analysis. Based on the above literature and SCT, the following hypotheses were proposed.

H1 Internship environment is positively associated with voice behavior.
H2 A significant indirect effect exists between internship environment and voice behavior through self-efficacy.
H3 A significant indirect effect exists between internship environment and voice behavior through professional identity.
H4 A significant combined indirect effect exists between internship environment and voice behavior involving self-efficacy and professional identity in the hypothesized order.

## 2 Methods

### 2.1 Study design and participants

This study was reported in accordance with the Strengthening the Reporting of Observational Studies in Epidemiology (STROBE) guidelines (von Elm et al., 2007). This multicenter cross-sectional study was conducted in three tertiary hospitals in Hunan Province, China. The three hospitals were tertiary teaching hospitals that routinely receive nursing interns for clinical training. Nursing interns from different clinical departments were invited to participate during their internship rotation. Convenience sampling was adopted, and nursing interns who were undergoing clinical internships during the research period were invited to participate in the survey. Participants were eligible if they were currently engaged in clinical internship and were able to complete the questionnaire independently. Interns who were absent during the survey period or who submitted incomplete questionnaires were excluded. Sample size was calculated using G^*^Power 3.1.9.7 software (Faul et al., 2007). As no directly comparable previous study was available to provide an expected effect size, a medium effect size was assumed according to Cohen's convention for multiple regression analysis (*f* ^2^ = 0.15) (Cohen, 1998). With α = 0.05, 1-β = 0.95, and 16 predictors, the minimum required sample size was 204. After allowing for a potential invalid response rate of 10%, at least 227 participants were required. Finally, 498 valid questionnaires were included in the analysis.

### 2.2 Instruments

#### 2.2.1 General information questionnaire

A self designed general information questionnaire was used to collect demographic and professional characteristics of the participants. The questionnaire included gender, age, education level, household registration, only child status, internship duration, experience as a student cadre, and perceived internship stress.

#### 2.2.2 Self-efficacy

The General Self-Efficacy Scale (GSES) was developed by German psychologist Schwarzer et al. (1997) and translated and revised by Chinese scholars Wang et al. (2001). This scale is a single-dimensional scale consisting of 10 items. It uses the Likert 4-point rating method, scoring from “completely incorrect” to “completely correct” with values ranging from 1 to 4. The higher the total score, the better the self-efficacy. In this study, the Cronbach's α coefficient for the total scale was 0.901.

#### 2.2.3 Professional identity

The questionnaire on professional identity of nursing students was developed by Hao (2011). It consists of 17 items, covering five dimensions: professional self-concept, benefits of retention and risks of resignation, social comparison and self-reflection, autonomy in career choice, and social persuasion. It uses the Likert 5-level scoring method, with 1 to 5 points assigned from “completely inconsistent” to “completely consistent,” and the total score ranges from 17 to 85 points. The higher the score, the more the nursing students identify with the nursing profession. In this study, the Cronbach's α coefficient for the total scale was 0.956.

#### 2.2.4 Internship environment

The Clinical Practice Environment Scale was developed by Chu (2010) and consists of 6 dimensions and 30 items, namely teaching methods, teacher quality, learning opportunities, interpersonal relationships, working atmosphere and organizational support, each with 5 items. The Likert 5-point scoring method was adopted, with 1 to 5 points assigned from “completely disagree” to “completely agree,” totaling 150 points. The higher the score, the better the clinical internship environment. In this study, the Cronbach's α coefficient for the total scale was 0.961.

#### 2.2.5 Voice behavior

The Voice Behavior Scale was developed by Liang et al. (2012). The scale consists of 10 items and two dimensions: promotive voice and prohibitive voice. The Likert 5-point rating system was adopted, with ratings ranging from “very inconsistent” (1 point) to “very consistent” (5 points). The higher the score, the stronger the willingness to make suggestions. In this study, the Cronbach's α coefficient for the total scale was 0.927.

### 2.3 Data collection

Data were collected from January to February 2026. After permission was obtained from the participating hospitals, nursing interns who met the inclusion criteria were invited to participate in the survey. The purpose of the research was explained to all participants before distributing the questionnaires. Participation was voluntary and anonymous. The questionnaires were distributed either in paper form or through an online survey platform. Each participant was allowed to submit the questionnaire only once. Questionnaires with missing key variables or evidently incomplete responses were excluded.

### 2.4 Statistical analysis

Statistical analyses were conducted using SPSS 27.0 (IBM, 2020). The distributions of the main continuous variables were assessed using skewness and kurtosis before Pearson correlation analysis. Descriptive statistics were used to summarize participants' demographic characteristics, with continuous variables presented as mean and standard deviation and categorical variables presented as frequencies and percentages. Harman's single-factor test was conducted to assess potential common method bias, and variance inflation factors (VIFs) were examined to assess multicollinearity among the study variables. Pearson correlation analysis was performed to examine the relationships among internship environment, self-efficacy, professional identity, and voice behavior. Regression analyses were conducted to estimate the path coefficients in the mediation models. The mediating effects of self-efficacy and professional identity were further examined using the PROCESS macro (Model 6) with 5,000 bootstrap resamples to estimate indirect effects and their 95% confidence intervals. A mediation effect was considered significant if the confidence interval did not include zero. For descriptive purposes, the ratio of the total indirect effect to the total effect was calculated as: total indirect effect/total effect × 100%. Given the cross-sectional design, this ratio was not interpreted as the proportion of a causal effect explained by the mediators. *P* < 0.05 was considered statistically significant.

### 2.5 Ethics statement

This study was approved by the Institutional Review Board of the Second Xiangya Hospital of Central South University (LYEC2026-K0033). All participants provided written informed consent before participation. The study was conducted in accordance with the Declaration of Helsinki.

## 3 Results

### 3.1 Sample characteristics

A total of 520 questionnaires were distributed to nursing interns. After excluding incomplete responses, 498 valid questionnaires were included in the final analysis, resulting in a response rate of 95.8%. The recruitment flowchart is shown in Figure 1.

**Figure 1:**
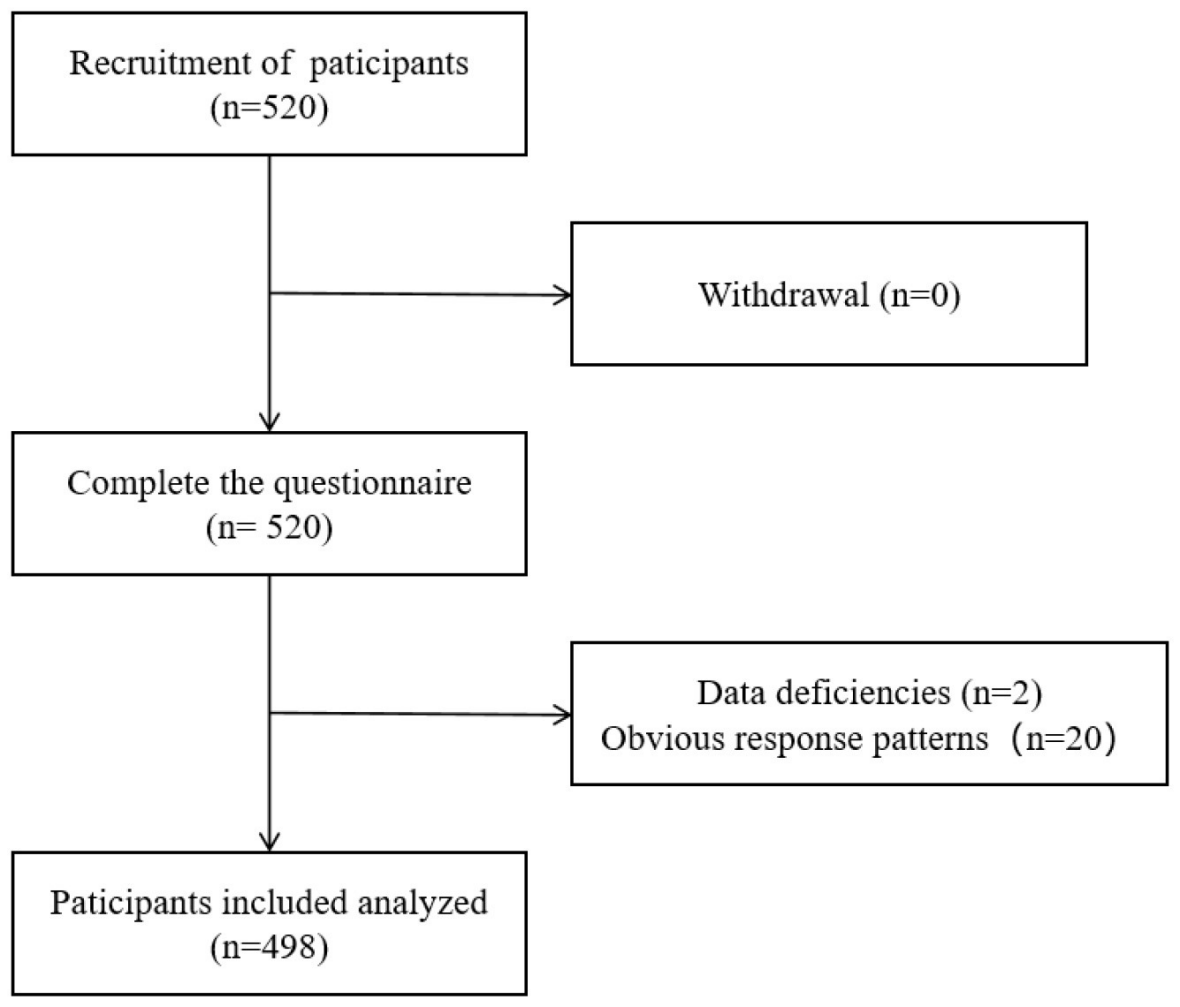
Flow diagram of participant recruitment and inclusion.

The mean age of the participants was 21.09 years (SD =1.38). Most participants were female (85.1%), and over half reported a moderate level of perceived internship stress (56.8%). Detailed information of demographic and professional characteristics is shown in Table 1.

**Table 1:** Demographic and professional characteristics of the study sample.

Variables	*N* (%)/*M* (SD)
Gender	
Female	424(85.1)
Male	74(14.9)
Age	21.09 ± 1.38
Education	
Vocational training	230(46.2)
Bachelor's Degree	239(48.0)
Master's Degree	29(5.8)
Household registration	
City	134(26.9)
Village	364(73.1)
Only child	
Yes	84(16.9)
No	414(83.1)
Internship duration	
<3 months	111(22.3)
3~6months	80(16.1)
>6months	307(61.6)
Experience as a student leader	
Yes	252(50.6)
No	246(49.4)
Self-assessment of perceived internship stress	
Very low	24(4.8)
Relatively low	42(8.4)
Average	283(56.8)
Relatively high	133(26.7)
Very high	16(3.2)

### 3.2 Pearson correlation analysis

The main continuous variables were approximately normally distributed, with skewness values ranging from −0.582 to −0.007 and excess kurtosis values ranging from −0.193 to 0.693. As shown in Table 2, internship environment was positively correlated with voice behavior (*r* = 0.553, *P* < 0.001), self-efficacy (*r* = 0.498, *P* < 0.001), and professional identity (*r* = 0.538, *P* < 0.001). Self-efficacy was positively associated with professional identity (*r* = 0.732, *P* < 0.001) and voice behavior (*r* = 0.639, *P* < 0.001). In addition, professional identity was positively correlated with voice behavior (*r* = 0.678, *P* < 0.001).

**Table 2:** Pearson correlations among internship environment, self-efficacy, professional identity, and voice behavior.

Variables	Voice behavior	Self-efficacy	Professional identity	Internship environment
voice behavior	1.000			
self-efficacy	0.639^**^	1.000		
professional identity	0.678^**^	0.732^**^	1.000	
internship environment	0.553^**^	0.498^**^	0.538^**^	1.000

^**^p < 0.001, indicating significance in correlation.

### 3.3 Mediation analysis

The results of Harman single factor test showed that the first factor explained 31.72% of the total variance, which was lower than the 40% threshold recommended by Podsakoff et al. (2003). In addition, VIF values across the adjusted regression models ranged from 1.037 to 2.479, indicating no serious multicollinearity.

An indirect-effect analysis was conducted using PROCESS Model 6 to estimate the individual and combined indirect effects involving self-efficacy and professional identity in the association between the internship environment and voice behavior. All models were adjusted for gender, age, education, residence, only-child status, internship duration, student leadership experience, and internship stress. The adjusted regression results are presented in Table 3, and the estimated indirect effects are presented in Table 4 and Figure 2.

**Table 3:** Regression results for voice behavior, promotive voice, and prohibitive voice.

Variables	Self-efficacy		Professional identity		Voice behavior	
	β	*t*	β	*t*	β	*t*
Model 1						
internship environment	0.513	12.834^**^	0.263	7.513^**^	0.258	6.672^**^
self-efficacy			0.596	17.316^**^	0.250	5.459^**^
professional identity					0.352	7.387^**^
*R^2^*	0.293		0.597		0.561	
*F*	14.261^**^		47.530^**^		38.360^**^	
Model 2						
internship environment	0.513	12.834^**^	0.263	7.513^**^	0.277	6.380^**^
self-efficacy			0.596	17.316^**^	0.176	3.432^**^
professional identity					0.319	5.960^**^
*R^2^*	0.293		0.597		0.446	
*F*	14.261^**^		47.530^**^		24.151^**^	
Model 3						
internship environment	0.513	12.834^**^	0.263	7.513^**^	0.208	5.368^**^
self-efficacy			0.596	17.316^**^	0.294	6.415^**^
professional identity					0.343	7.191^**^
*R^2^*	0.293		0.597		0.559	
*F*	14.261^**^		47.530^**^		38.155^**^	

β = standardized regression coefficient; ^**^p < 0.001.

**Table 4:** Estimated indirect effects involving self-efficacy and professional identity in the association between the internship environment and voice behavior.

Path	Effect value	Boot SE	Descriptive ratio (%)	LLCI	ULCI
Model 1					
Internship environment → self-efficacy → voice behavior	0.050	0.011	21.88	0.027	0.071
internship environment → professional identity → voice behavior	0.036	0.007	15.77	0.023	0.051
internship environment → self-efficacy → professional identity → voice behavior	0.042	0.008	18.32	0.027	0.058
Direct effect	0.100	0.015	43.98	0.071	0.130
Total indirect effect	0.127	0.013	55.98	0.102	0.152
Total effect	0.228	0.015	100.00	0.199	0.257
Model 2					
internship environment → self-efficacy → Promotive voice	0.019	0.007	16.52	0.006	0.032
internship environment → professional identity → Promotive voice	0.017	0.004	15.29	0.010	0.026
internship environment → self-efficacy → professional identity → Promotive voice	0.020	0.005	17.75	0.011	0.030
Direct effect	0.057	0.009	50.44	0.040	0.075
Total indirect effect	0.056	0.008	49.47	0.041	0.071
Total effect	0.114	0.008	100.00	0.098	0.130
Model 3					
internship environment → self-efficacy → Prohibitive voice	0.031	0.006	27.24	0.020	0.042
internship environment → professional identity → Prohibitive voice	0.019	0.004	16.26	0.012	0.026
internship environment → self-efficacy → professional identity → Prohibitive voice	0.022	0.004	18.89	0.014	0.030
Direct effect	0.043	0.008	37.52	0.027	0.058
Total indirect effect	0.071	0.007	62.48	0.058	0.084
Total effect	0.114	0.008	100.00	0.098	0.130

All models were adjusted for gender, age, education, residence, only-child status, internship duration, student leadership experience, and internship stress. The ratio was calculated as the total indirect effect divided by the total effect and multiplied by 100. It is reported descriptively and should not be interpreted as the proportion of a causal effect explained by the mediators.

**Figure 2:**
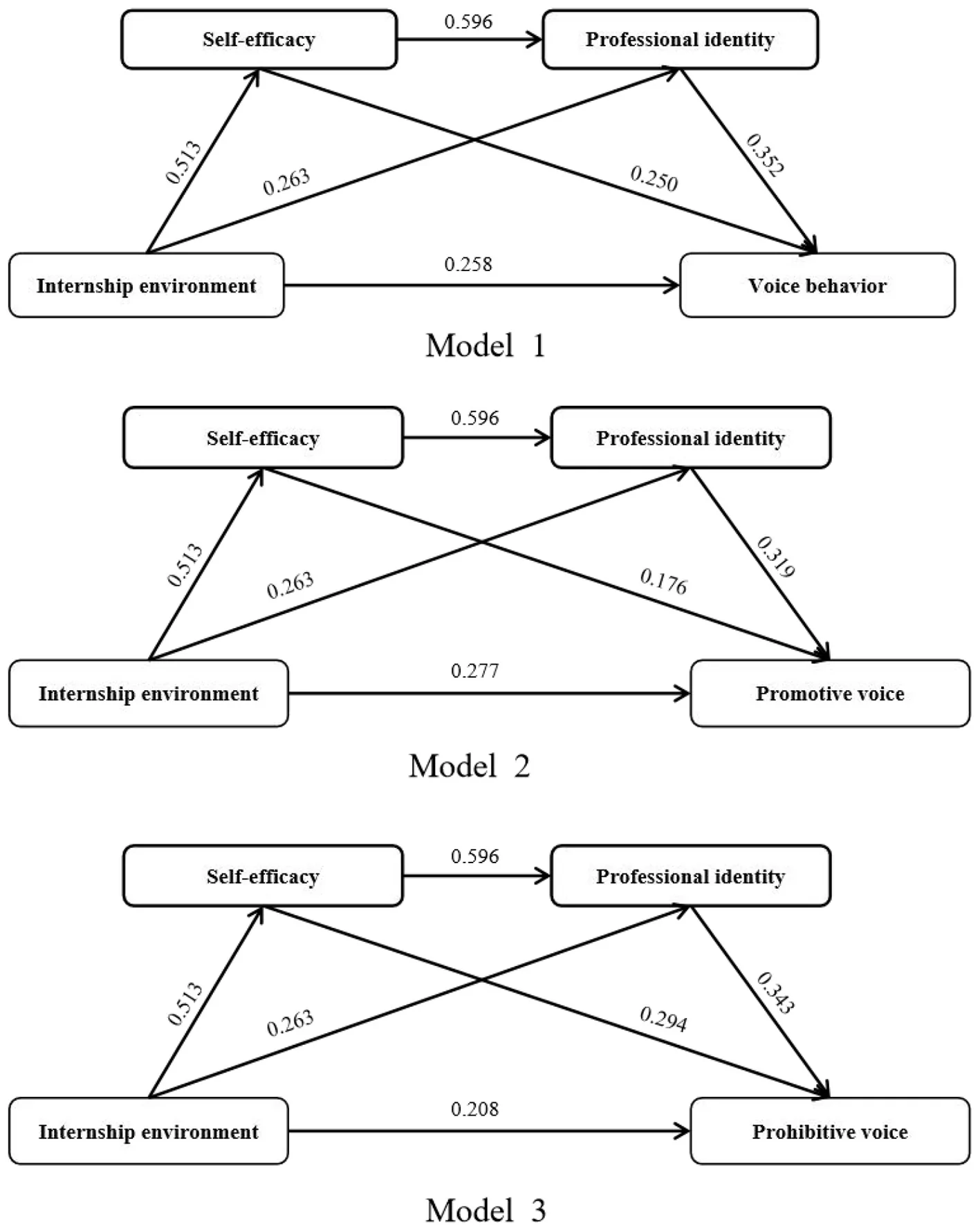
Statistical models of the indirect effects involving self-efficacy and professional identity in the association between the internship environment and voice behavior. Values represent standardized regression coefficients.

For overall voice behavior, the estimated total effect of the internship environment was significant [*B* = 0.228, 95% *CI* (0.199, 0.257)], and the estimated direct effect remained significant after self-efficacy, professional identity, and the covariates were included in the model [*B* = 0.100, 95% *CI* (0.071, 0.130)]. The 95% bootstrap confidence intervals did not include zero for the indirect effect involving self-efficacy [*B* = 0.050, 95% bootstrap *CI* (0.027, 0.071)], the indirect effect involving professional identity (*B*=0.036, 95% bootstrap *CI* [0.023, 0.051]), and the combined indirect effect involving both self-efficacy and professional identity [*B* = 0.042, 95% bootstrap *CI* (0.027, 0.058)]. The descriptive ratios of the estimated total indirect effect to the estimated total effect were 55.98% for overall voice behavior, 49.47% for promotive voice, and 62.48% for prohibitive voice.

## 4 Discussion

This study found that a more supportive internship environment was associated with greater overall, promotive, and prohibitive voice behavior among nursing interns. The significant indirect effects involving self-efficacy and professional identity, both separately and jointly, suggest that voice behavior is related not only to the clinical learning environment but also to interns' confidence in their abilities and identification with the nursing profession. This pattern is consistent with SCT, which emphasizes the interrelationships among environmental factors, personal factors, and behavior.

Speaking up can be particularly challenging for nursing interns because hierarchical clinical environments, authority gradients, and fear of negative evaluation may discourage them from questioning established practices or raising patient safety concerns (Hoffmann et al., 2022). When nursing interns perceive their learning environment as supportive and inclusive, they may feel more psychologically safe and therefore more willing to express suggestions or raise concerns related to patient care.

Self-efficacy may help explain the observed association between the internship environment and voice behavior. According to SCT, individuals' beliefs in their capabilities are closely related to their behavioral choices and persistence (Bandura, 2001). Supportive supervision, constructive feedback, and successful clinical experiences may be associated with greater confidence in recognizing problems, formulating suggestions, and communicating with senior staff. This interpretation is consistent with previous evidence linking higher self-efficacy to greater engagement in proactive behaviors (Jiang et al., 2023; El-Sayed et al., 2025). Thus, self-efficacy may represent an important psychological correlate of nursing interns' willingness to speak up.

Professional identity may be related to voice behavior in a different but complementary way. Previous studies have suggested that individuals with stronger professional identity tend to demonstrate higher levels of professional commitment and engagement (Sarraf-Yazdi et al., 2024; Clements et al., 2016). Nursing interns who strongly identify with the nursing profession may feel a greater responsibility for patient safety and quality improvement. This sense of responsibility may motivate them to express ideas or concerns when they observe potential problems in clinical practice. Thus, professional identity may provide a motivational and ethical basis for speaking up, complementing the perceived capability reflected in self-efficacy.

The combined indirect effect involving self-efficacy and professional identity may reflect the complementary relevance of perceived capability and professional responsibility to voice behavior. Self-efficacy may be associated with interns' confidence in expressing their views, whereas professional identity may be associated with their motivation to raise issues concerning patient safety and care quality (Huang et al., 2025; Fagan et al., 2016). This pattern is consistent with SCT, which emphasizes the interrelationships among environmental context, personal cognitive factors, and behavior (Bandura, 2001). The indirect effect was descriptively larger for prohibitive voice than for promotive voice. Because prohibitive voice involves identifying risks, errors, or harmful practices, it may be perceived as more interpersonally challenging in hierarchical clinical settings (Lainidi et al., 2023). Therefore, nursing interns may require stronger psychological resources to engage in this type of voice behavior (Yang et al., 2025).

From a practical perspective, these findings highlight the importance of creating supportive internship environments in clinical education. Nursing educators and clinical supervisors should consider establishing clear channels for reporting concerns, responding constructively to interns' suggestions, and providing regular feedback and mentorship. Training in assertive communication and patient safety may further strengthen interns' confidence in raising concerns, while clinical mentorship and professional role modeling may support the development of their professional identity (Hanson et al., 2020). These measures may help create a clinical learning environment in which nursing interns perceive voice behavior as both acceptable and professionally meaningful.

Several limitations should be acknowledged. First, because all variables were measured concurrently, the cross-sectional data cannot establish temporal ordering or causal relationships among the internship environment, self-efficacy, professional identity, and voice behavior. Second, although the survey was anonymous and Harman's single-factor test was conducted, common method bias cannot be completely excluded because all variables were measured using self-reports at the same time. Third, although demographic and internship-related characteristics were controlled, other potentially relevant factors, such as psychological safety, supervisory support, and organizational climate, were not measured. Future longitudinal studies could examine these factors across more diverse clinical settings and populations.

## 5 Conclusion

This study found that internship environment was positively associated with nursing interns' voice behavior, including promotive and prohibitive voice. Significant individual and combined indirect effects involving self-efficacy and professional identity were also observed. Clinical educators may consider providing constructive feedback and professional role modeling to support interns' confidence and professional development. Clear and psychologically safe channels for expressing suggestions and patient safety concerns should also be established.

## Data Availability

The raw data supporting the conclusions of this article will be made available by the authors, without undue reservation.
